# SpacED POCUS: A Randomized Controlled Trial of an Adaptive Spaced Education POCUS Curriculum for Medical Students

**DOI:** 10.24908/pocusj.v10i02.19092

**Published:** 2025-11-17

**Authors:** Anelah McGinness, Sarah Hancock, Megan Hilbert, Jane Soung, Emily Lovallo

**Affiliations:** 1 University of Pittsburgh Medical Center; 2 University of Pittsburgh School of Medicine

**Keywords:** POCUS education, Point of care ultrasound, Online POCUS education, Retention, Spaced education, Medical education

## Abstract

This study evaluated the effectiveness, retention, engagement, and acceptability of adaptive spaced education (spacED) for improving the accuracy of point of care ultrasound (POCUS) image interpretation by medical students. From July 2022-May 2023, students (n=36) were randomized into two groups and each assigned 50 unique POCUS cases: cardiac/vascular or lung/FAST. Each one served as the control for the other group. We measured effectiveness (% posttest 1 – % pretest), six-month retention (% posttest 2 – % posttest 1), engagement (% cases completed), and acceptability (% would recommend). Twenty-nine students (81%) completed the study. On average, 38.6% of cases were completed over the six-month study period. There was a significant increase in test scores covering FAST (Focused Assessment with Sonography in Trauma) (+18%), lung (+25%), and vascular (+23%, all p<0.01). Six-month FAST and lung scores did not have significant loss (+3% and –10%, p >0.05). Acceptability was high; 96% of students indicated they would participate again. Despite an imperfect case completion rate, for some applications, spacED was an effective, long-lasting, and acceptable method for teaching POCUS interpretation to medical students.

## Introduction

Adaptive spaced education (spacED) is an evidence-based, asynchronous teaching method that encodes new knowledge through repeated exposure to content over time [1]. SpacED uses principles of retrieval practice and interleaving to achieve gains in knowledge that are long lasting and associated with changes in clinical behavior [2–5]. Adaptive methods optimize efficiency by bringing unmastered content to the front and discarding mastered content, thus maximizing learning with minimal questions [1]. While adaptive spacED has been used successfully for radiology curricula, it has yet to be explored as a tool for teaching the interpretation of point of care ultrasound (POCUS) images [4,6,7].

We piloted an asynchronous, online 12-month spacED POCUS curriculum focused on POCUS interpretation for third-year medical students engaged in clinical clerkships. In this study, we measured the effectiveness of our adaptive spacED curriculum, with follow up evaluation six months later to assess retention. We also performed subgroup analysis to examine the effect of engagement and previous POCUS exposure. To assess feasibility, we reported on both the engagement and acceptability of the course.

## Methods

Of 180 students in the class, 36 third-year medical students volunteered to participate in this study as an ungraded elective concurrently with their clinical rotations. Using results from prior spacED studies, we calculated that a sample size of 50 students would achieve a power of 0.8 to detect an effect size of 15% and a variance of 30% with a 2-sided 0.05 significance level [3,4,6].

A subset of students at our institution completed a longitudinal ultrasound “POCUS Certificate Program” from their second to fourth years of medical school. In preclinical years, content focused on image acquisition. In the fourth year, certificate students completed a clinical POCUS elective with scanning shifts and near-peer teaching. A noticeable loss of knowledge seemed to occur during the gap in the third year when in-person POCUS education was limited due to conflicting clinical schedules.

### Design

**Figure 1.**
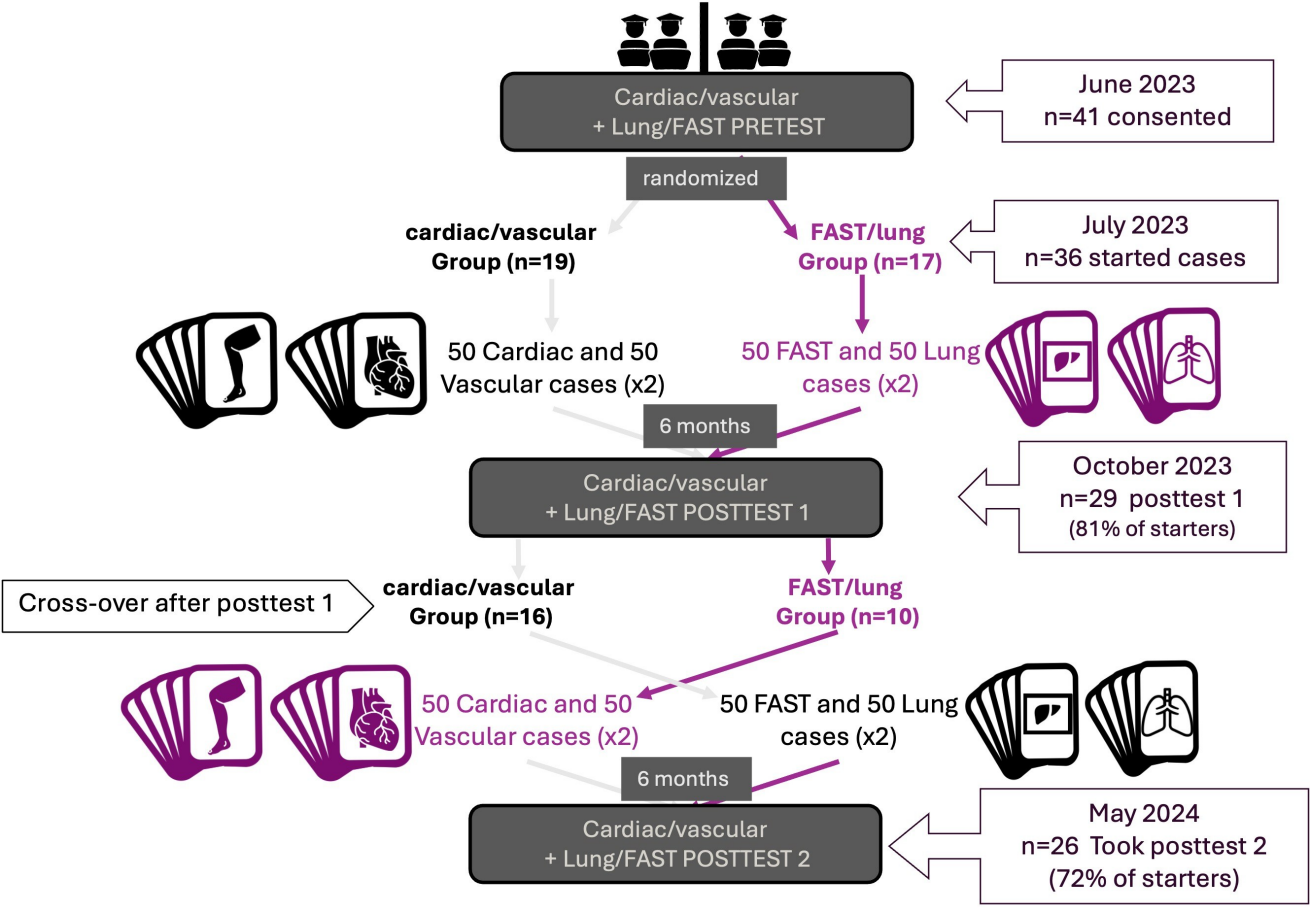
Overview of course structure and sampling. FAST: Focused Assessment with Sonography in Trauma.

The structure of our course is detailed in Figure 1. We divided the content into four chapters each consisting of 50 cases: cardiac, vascular, Focused Assessment with Sonography in Trauma (FAST), and lung. All participants took the same forty item pretest which included ten questions in each of the content chapters. Students were randomized into two separate groups: a FAST/lung group and a cardiac/vascular group. Two of the authors (AM, SH) were course administrators and not blinded to participant assignments.

**Figure 2.**
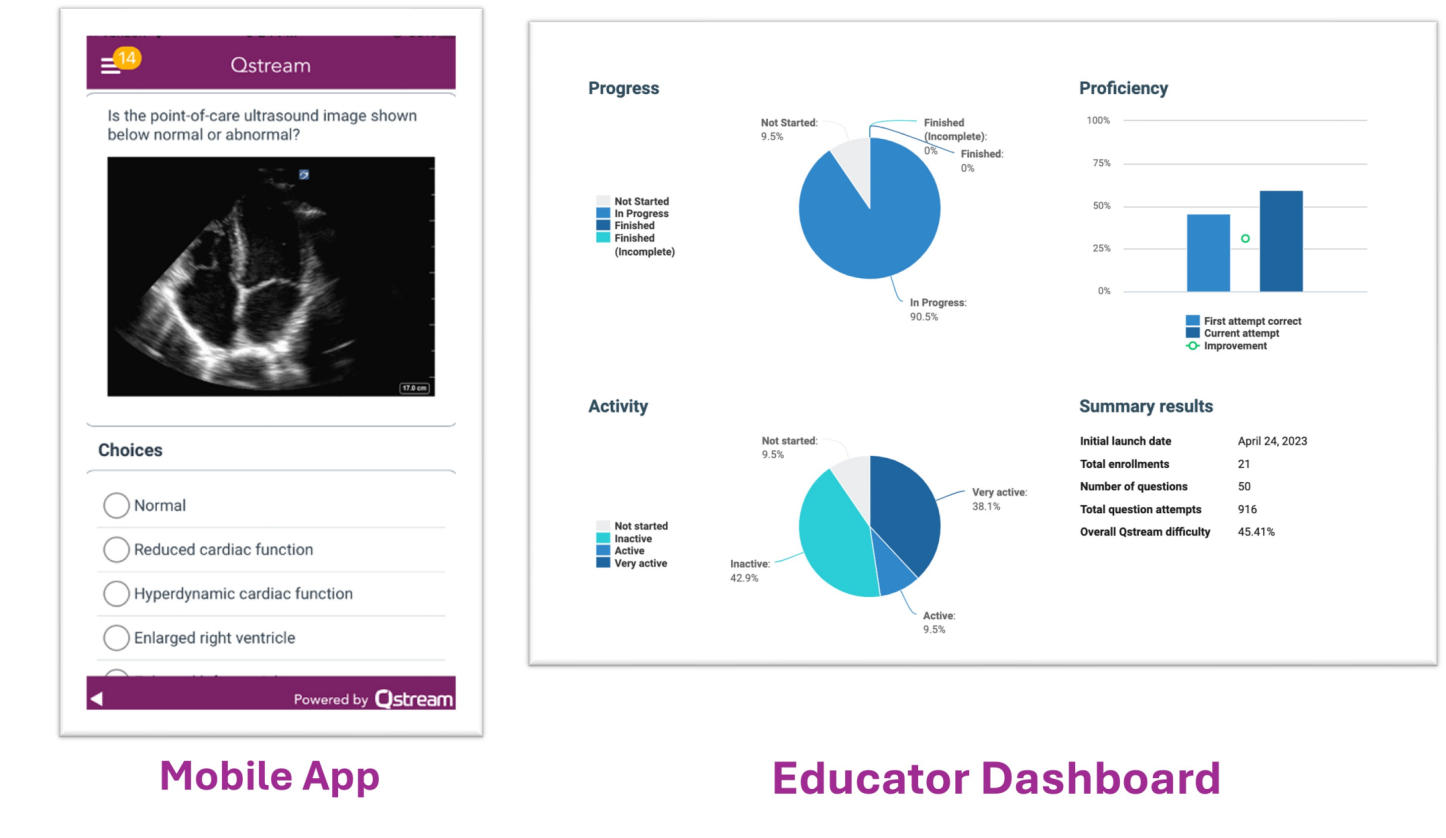
Screenshots of the spaced education (spacED) platform mobile application and desktop educator dashboard.

For six months, students interpreted either 100 unique FAST and lung cases (50 each) or 100 unique cardiac and vascular cases (50 each). Students were sent three cases from each chapter every three days, excluding weekends. Correctly interpreted cases were repeated in two weeks, while incorrect cases were repeated in one week. When students interpreted the same case correctly twice, it was retired and not re-sent. Following established recommendations from prior research regarding the optimal ratio of pathology, we presented students with 50% normal and 50% pathologic videos [8–10]. A sample case is shown in Figure 2. Each student viewed a minimum of 200 cases between their two chapters, consistent with prior studies on radiology and POCUS learning curves [8–10]. To prevent question fatigue, cases that were incorrectly answered three times were retired. We recorded the mean percentage of cases retired as a measure of engagement.

At six-months, we reviewed commonly missed cases during evening image review sessions (1 hour in length). All students then completed the first posttest (posttest 1), which again covered all four content areas. Each group served as a maturation control for the other group. For the second half of the clinical year, we released the lung and FAST chapters to students in the former cardiac/vascular group, and the cardiac and vascular chapters to the former lung/FAST group. To measure six-month retention, we conducted a final evening review session and a second posttest at the end of the clinical year, which was six months from the first posttest.

The course was administered via Qstream (Burlington, MA), which is a spacED platform with a desktop website and mobile application (Figure 2). Through Qstream, students viewed animated clips (.gifs) of a POCUS study. They interpreted the case as either normal or selected “all that apply” from a list of pathologies (Appendix A). Students received immediate feedback with answers and explanations prior to moving on to the next case.

### Content Validity Analysis for Selection of Cases

The selected pathologies were based on the objectives and consensus standards from the American College of Emergency Physicians [11–12]. All cases and questions were reviewed and edited by all authors (AM, SH, MH, EL), two of whom are experts in POCUS education (MH, EL). Case content was stripped of patient identifiers.

### Data Analysis

Using Prism software (Irvine, CA), we conducted the Mann-Whitney-U test to compare intervention (median of intervention paired % posttest 1 - % pretest) to control groups (median of control paired % posttest 1 - % pretest). We used the Wilcoxon matched-pairs signed-rank test for paired data to test six-month retention (six-month follow up test % posttest 2 - % posttest 1). We used Mann-Whitney-U to compare subgroups among the students.

We measured engagement as the mean percentage of cases retired by students. We measured acceptability as the percentage of students who would take the course again and the percentage who would recommend the course to others.

## Results

### Participant Characteristics and Engagement

Demographic information is summarized in Table 1. Overall, demographics and prior POCUS education were similar between groups. Of the 41 medical students who consented to participate in spacED POCUS, 36 students initiated the program. Among these, 29 (81%) completed posttest 1. The average student completed 38.6% of cases (∼39 out of 100 cases). A total of 11 students (27%) achieved completion of all assigned cases, with 24% (4/17) of students completing the FAST and lung cases and 32% (7/19) completing the cardiac and vascular cases.

**Table 1.** Participant demographics. POCUS: Point of care ultrasound. FAST: Focused Assessment with Sonography in Trauma.

Randomized group assignment	Cardiac/vascular	FAST/lung	Significance
	N (percent of group)	N (percent of group)	(P)
Count *(n)*	19	18	
Average age *(years, 95% CI)*	25.4 (24.3 – 26.5)	25.3 (24.1 – 26.4)	0.374
Underrepresented in medicine	4 (21.1%)	4 (22%)	1.0
**Gender**			
Female	11 (57.9%)	10 (55.6%)	1.0
Male	7 (36.8%)	6 (33.3%)	1.0
Otherwise specified	1 (5.3%)	2 (3.8%)	0.60
**Previous POCUS education**			
Longitudinal curriculum	9 (47.5%)	8 (44.4%)	1.0
Workshop(s)	9 (52.6%)	7 (38.9%)	0.74
None	0 (0%)	3 (16.7%)	0.105
**Estimated educational POCUS scans performed**			
<25 scans	15 (78.9%)	14 (73.7%)	1.0
25 – 50 scans	4 (21.1%)	4 (21.1%)	1.0
50–100 scans	0 (0%)	1 (5.3%)	0.49

### Knowledge Acquisition and Test Performance

**Figure 3.**
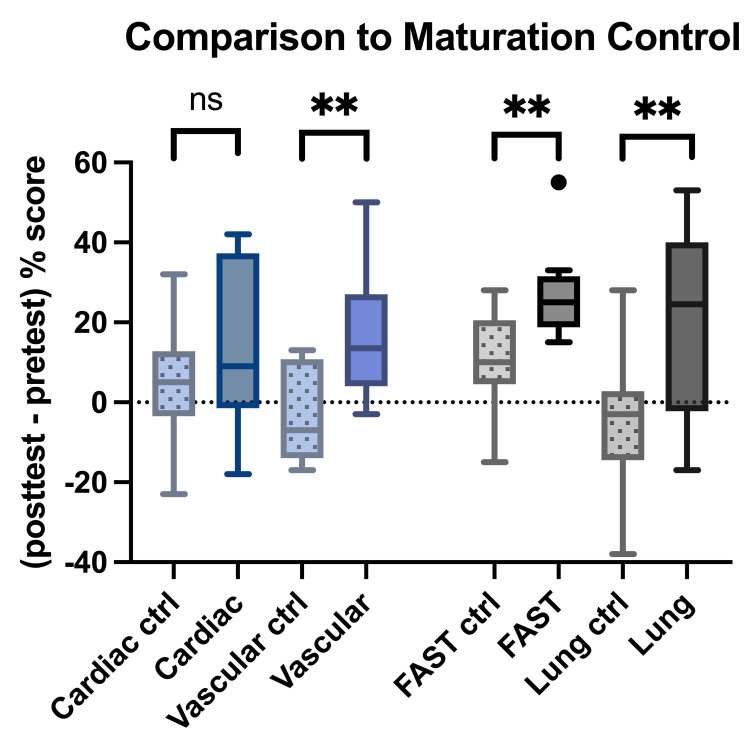
Change in median point of care ultrasound (POCUS) interpretation test scores, significance when compared to maturation controls. Effectiveness was measured as (posttest 1 – pretest) score for each student. Changes in score for each student are represented by plots above. ** denotes p-value <0.01 ns denotes p-value >0.05. FAST: Focused Assessment with Sonography in Trauma.

Student test scores showed significant improvement in the vascular, FAST, and lung test scores with p<0.05 (paired % posttest 1 – % pretest) (Table 2). All remained statistically significant (p <0.01) when compared to maturation controls, except for the cardiac chapter (p=0.30) (Figure 3).

**Table 2.** Median test scores. FAST: Focused Assessment with Sonography in Trauma.+ 6 months after posttest 1; ++ Median of all students' improvement from pretest to posttest 1

		Pretest	Posttest 1	Posttest 2 +	Median Improvement After Intervention ++
	Count (n)	Median Score % (95% CI)	Median Score % (95% CI)	Median Score % (95% CI)	Change in Median Score % (95% CI)
**Cardiac**					
Control	10	35.0 (25.0, 75.0)	50.0 (23.0, 60.0)	61.5 (53.0, 83.0)	+5.0 (−5.0, 15.6)
Intervention	16	45.0 (35.0, 65.0)	65.0 (60.0, 77.0)	63.0 (43.0, 73.0)	+9.0 (3.2, 24.6)
					P = 0.30
**Vascular**					
Control	10	40.0 (20.0, 50)	33.0 (27.0, 53.0)	33.0 (20.0, 53.0)	−7.0 (−11.2, 6.2)
Intervention	16	40.0 (30.0, 50.0)	63.5 (53, 80)	47.0 (20, 53)	+13.5 (9.2, 26.0)
					P <0.01
**FAST**					
Control	16	50.0 (45.0, 70.0)	52.5 (39.0, 73.0)	63.0 (51.0, 78.0)	+10.0 (3.4, 16.6)
Intervention	10	45.0 (30.0, 55.0)	63.0 (59.0, 87.0)	65.5 (59.0, 85.0)	+26.7 (18.5, 35.0)
					P <0.01
**Lung**					
Control	16	50.0 (25.0, 65.0)	40.0 (27.0, 53.0)	60.0 (53.0, 80)	−3.0 (−15.7, 3.0)
Intervention	10	35.0 (20.0, 40.0)	60.0 (33.0, 73.0)	50.0 (40.0, 80.0)	+24.5 (4.6, 36.2)
					P <0.01

### Subgroup analysis

The students who retired all 100 of their assigned cases, or “completionists,” did not learn more when compared to their remaining group members. Completionists (n=11) showed a median improvement (paired % posttest 1 - % pretest) of +17% in their assigned content, while the non-completionists showed a median improvement of +18% (p=0.78). Completionists also did not perform better on the curriculum training material. The completionists got 59% of their assigned Qstream cases correct twice, meaning that the remaining 41% of cases were retired because they were answered incorrectly three times. Students who did not complete all assigned cases retired 55% of the cases from correctly answering twice.

Of the 36 total students, 17 had previous POCUS exposure through the POCUS Certificate Program. The certificate students showed a median improvement of +19% in their assigned content while the noncertificate students showed a similar median improvement of +17% (p=0.67).

### Retention at Six Months

**Figure 4.**
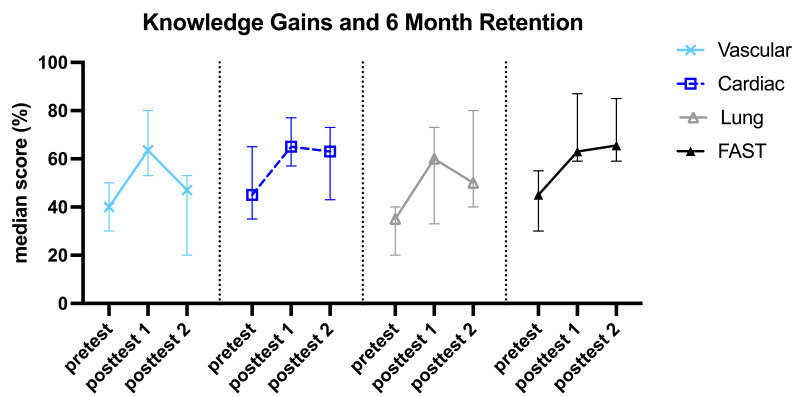
Median increases in knowledge (pretest to posttest 1) and 6-month retention (posttest 2). Data points represent median test scores. Error bars represent 95% confidence intervals. FAST: Focused Assessment with Sonography in Trauma.

At the six-month follow-up, we assessed knowledge retention (paired posttest 2 % - posttest 1 %). See Figure 4. The FAST (+3%), lung (-10%), and cardiac (-1.67%) groups maintained median test scores similar to their first posttest, which did not demonstrate a statistically significant reduction in scores on paired analysis. There was, however, a significant decrease in the median retention of vascular content (-16.7%, p<0.01). Interpreting retention was limited for the cardiac group as there was already no significant improvement from the intervention between the pretest and first posttest (Figure 3).

### Student Feedback and Acceptability

The program was well reviewed by students with 96% indicating they would participate in a spacED curriculum again and 100% recommending the course to other learners. Qualitative feedback highlighted the usefulness of the longitudinal format, where students cited examples of applying the material during clinical rotations. Students suggested adding initial orientation meetings, frequent review sessions, and the ability to customize the spacing interval for new and repeated questions.

## Discussion

In this pilot study, we developed an asynchronous curriculum that utilized the adaptive spacED model to improve POCUS image interpretation accuracy and aid in long-term retention of these skills. We demonstrated statistically significant improvement in all topics (vascular, FAST, and lung), except for the cardiac chapter when compared to the control group.

The lack of improvement in cardiac cases is consistent with previous studies, which have also found that the interpretation of cardiac POCUS may be more challenging relative to other topics [9,10]. Although we were able to see an improvement in accuracy for the non-cardiac topics ranging from 18% to 25%, we did not hold students to a competency standard to pass because this was an elective course.

Our high retention for the FAST and lung content was consistent with previous studies showing that adaptive spacED is associated with increased long-term retention. Previous studies have used identical questions in their tests, which may introduce image recall or memorization rather than knowledge acquisition [2]. We intentionally used a mix of novel and repeated cases in our posttests to ensure our assessments were of comparable difficulty while allowing our students to apply their skills to novel content.

A study by Mathes et al. had contrary results to our pilot. They failed to show significant knowledge gain in pediatric dermatologic conditions when using non-adaptive spacED [13]. This may be due to a relatively low number of cases (12 items) assigned for each topic. Kwan et al. suggested that the number of POCUS cases to obtain 90% accuracy of image interpretation ranged from 87 to 128 cases per content area [9,10]. Pusic et al. found that the highest rate of improvement in pediatric residents interpreting radiographs occurred between 21 and 50 cases with an inflection point of slowed learning at 234 cases [8]. Our curriculum required a minimum of 100 views per chapter. Overall, median posttest scores ranged 60-65%, and the average student completed 38 of 100 assigned unique cases. We do not know how much learning would have slowed after reaching 60% accuracy or how many students would have been able to reach an accuracy of 90% as in prior studies. If our course were required, the next steps would have been to determine the feasibility of implementing a competency-based standard.

Current competency guidelines for emergency medicine practitioners rely on number-based benchmarks, rather than performance-based measures [14]. Harel-Sterling et al. used a multi-phased approach to derive a performance-based competency standard for POCUS image interpretation [15]. Similarly, we focused specifically on POCUS image interpretation, but our study was not designed to prove the efficacy of adaptive spacED as a tool for competency.

Our data showed no difference in improvement between students who had completed the POCUS Certificate Program and those without additional POCUS education. The current second year Certificate Program curriculum focused on image acquisition and less on interpretation and pathologic findings. This may explain the lack of difference but also highlights that students with different levels of prior POCUS experience were able to improve accuracy.

Regarding acceptability, we found our adaptive spacED approach was positively received by the medical student participants. We had 96% of respondents reporting that they would participate in a similar course again and 100% said they would recommend this approach to others. As POCUS curricula continue to increase among medical schools, there has been overwhelming support from the trainees; however, almost all studies acknowledge the many barriers to implementing an integrated longitudinal curriculum spanning the pre-clinical and clinical years [16–19]. While 57% of medical doctorate-granting medical schools reported having a POCUS curriculum as of 2022, only 8% offered a longitudinal POCUS curriculum [20]. An asynchronous, adaptive curricula such as ours adjusts to the significant variation in schedules and academic requirements among clerkships while providing a benchmark that can be retested over time.

## Limitations

Our power was limited by sample size. We had only 36 students enrolled in our study, compared to the desired power calculation of 50 students. This number was further reduced once the included students were divided into groups. When asked, students who did not enroll responded that they had interest in learning POCUS but hesitated to enroll in a course prior to the transition to clinical clerkships.

We anticipated from this project's onset that implementing the intervention simultaneously with clinical rotations would be a challenge to feasibility. As with all educational assignments, asynchronous or not, adequate means of accountability and acceptance are necessary for motivating students to complete their assigned cases. Aside from a six-month deadline in the form of a posttest, this course was not required. Students predictably prioritized their clinical obligations. Even though only an average of 38.6% of all assigned cases were retired, 100% of students responded that they would recommend the course. However, we learned that perfect engagement is not necessary for significant gains in knowledge. Given that completionists did not have significantly higher test scores than non-completionists, we do not believe that mere repetition or completion is sufficient to achieve higher accuracy. Instead, we suggest that students achieve a defined competency standard.

We do not know the impact that our image review sessions may have had on posttest scores. In retrospect, testing prior to image review would eliminate this concern. The focus of image review was to correct common misconceptions.

While we aimed for slow, steady progression through the material, we received feedback that participants were more willing to review the material in larger chunks at a time as their schedules allowed. We standardized minimum spacing, but the optimal spacing and item load per session remains a question to be explored. To these authors' knowledge the optimal spacing of learning has not yet been elucidated.

## Conclusion

Overall, adaptive spacED was an effective tool for teaching POCUS interpretation with long-term retention and measurable knowledge acquisition. Even with imperfect engagement, this course was effective and well accepted by medical students during clinical rotations.

Future areas of study include trialing this method using an accuracy-based performance benchmark and determining the optimal settings for spacing to ensure knowledge acquisition and long-term retention.

## References

[1] Kerfoot BP. Adaptive Spaced Education Improves Learning Efficiency: A Randomized Controlled Trial. Journal of Urology 2010;183(2):678–681. doi:10.1016/j.juro.2009.10.005 20022032

[2] Kerfoot BP. Learning benefits of on-line spaced education persist for 2 years. The Journal of urology 2009;181(6):2671–2673. 19375095 10.1016/j.juro.2009.02.024

[3] Tshibwabwa E, Mallin R, Fraser M, Tshibwabwa M, Sanii R, Rice J, Cannon J. An Integrated Interactive-Spaced Education Radiology Curriculum for Preclinical Students. J Clin Imaging Sci 2017;24;7:22. doi: 10.4103/jcis.JCIS_1_17 PMC5450459 28584689

[4] Choe AI, Woodard S, Thompson BM, Walter V, Fotos JS, Kasales CJ. Spaced Education: Randomized Trial Comparing Learning Efficiency of the Adaptive Versus Nonadaptive Spaced Education Systems Among Radiology Residents. J Am Coll Radiol 2022;19(6):706–710. doi:10.1016/j.jacr.2022.03.010 35472369

[5] Shaw T, Long A, Chopra S, Kerfoot BP. Impact on clinical behavior of face-to-face continuing medical education blended with online spaced education: a randomized controlled trial. J Contin Educ Health Prof 2011;31(2):103–8. doi: 10.1002/chp.20113 21671276

[6] Morin CE, Hostetter JM, Jeudy J, Kim WG, McCabe JA, Merrow AC, Ropp AM, Shet NS, Sidhu AS, Kim JS. Spaced radiology: encouraging durable memory using spaced testing in pediatric radiology. Pediatr Radiol 2019;49(8):990–999. doi: 10.1007/s00247-019-04415-3 31093725 PMC6598954

[7] Thau E, Perez M, Pusic MV, Pecaric M, Rizzuti D, Boutis K. Image interpretation: Learning analytics–informed education opportunities. AEM Education and Training. 2021;5(2):e10592. doi:10.1002/aet2.10592 33898916 PMC8062270

[8] Pusic M, Pecaric M, Boutis K. How Much Practice Is Enough? Using Learning Curves to Assess the Deliberate Practice of Radiograph Interpretation. Academic Medicine 2011;86(6):731–736. doi:10.1097/acm.0b013e3182178c3c 21512374

[9] Kwan C, Weerdenburg K, Pusic M, Constantine E, Chen A, Rempell R, Herman JE, Boutis K. Learning Pediatric Point-of-Care Ultrasound: How Many Cases Does Mastery of Image Interpretation Take? Pediatr Emerg Care 2022;1;38(2):e849–e855. doi: 10.1097/PEC.0000000000002396 35100784

[10] Kwan C, Pusic M, Pecaric M, Weerdenburg K, Tessaro M, Boutis K. The Variable Journey in Learning to Interpret Pediatric Point-of-care Ultrasound Images: A Multicenter Prospective Cohort Study. AEM Education and Training 2020;4(2):111–122. doi:10.1002/aet2.10375 32313857 PMC7163207

[11] Physicians AC of E. Ultrasound Guidelines: Emergency, Point-of-Care and Clinical Ultrasound Guidelines in Medicine. Ann Emerg Med 2017;69(5):e27–e54. doi:10.1016/j.annemergmed.2016.08.457 28442101

[12] Marin JR, Abo AM, Arroyo AC, Doniger SJ, Fischer JW, Rempell R, Gary B, Holmes JF, Kessler DO, Lam SH, Levine MC, Levy JA, Murray A, Ng L, Noble VE, Ramirez-Schrempp D, Riley DC, Saul T, Shah V, Sivitz AB, Tay ET, Teng D, Chaudoin L, Tsung JW, Vieira RL, Vitberg YM, Lewiss RE. Pediatric emergency medicine point-of-care ultrasound: summary of the evidence. Crit Ultrasound J 2016;8(1):16. doi: 10.1186/s13089-016-0049-5. 27812885 PMC5095098

[13] Mathes EF, Frieden IJ, Cho CS, Boscardin CK. Randomized Controlled Trial of Spaced Education for Pediatric Residency Education. Journal of Graduate Medical Education 2022;6(2):270–274. doi:10.4300/jgme-d-13-00056.1 PMC4054725 24949130

[14] Abo AM, Alade KH, Rempell RG, Kessler D, Fischer JW, Lewiss RE, Raio CC, Marin JR. Credentialing Pediatric Emergency Medicine Faculty in Point-of-Care Ultrasound: Expert Guidelines. Pediatr Emerg Care. 2021;1;37(12):e1687–e1694. doi: 10.1097/PEC.0000000000001677 30624416

[15] Harel-Sterling M, Kwan C, Pirie J, Tessaro M, Cho DD, Coblentz A, Halabi M, Cohen E, Nield LE, Pusic M, Boutis K. Competency Standard Derivation for Point-of-Care Ultrasound Image Interpretation for Emergency Physicians. Ann Emerg Med 2023;81(4):413–426. doi: 10.1016/j.annemergmed.2022.11.002 36774204

[16] Minardi J, Ressetar H, Foreman T, Craig K, Sharon M, Bassler J, Davis S, Machi A, Cottrell S, Denne N, Ferrari N, Landreth K, Palmer B, Schaefer G, Tallaksen R, Wilks D, Williams D. Longitudinal Ultrasound Curriculum Incorporation at West Virginia University School of Medicine: A Description and Graduating Students' Perceptions. J Ultrasound Med 2019;38(1):63–72. doi: 10.1002/jum.14662 29732601

[17] Hoppmann RA, Rao VV, Bell F, Poston MB, Howe DB, Riffle S, Harris S, Riley R, McMahon C, Wilson LB, Blanck E, Richeson NA, Thomas LK, Hartman C, Neuffer FH, Keisler BD, Sims KM, Garber MD, Shuler CO, Blaivas M, Chillag SA, Wagner M, Barron K, Davis D, Wells JR, Kenney DJ, Hall JW, Bornemann PH, Schrift D, Hunt PS, Owens WB, Smith RS, Jackson AG, Hagon K, Wilson SP, Fowler SD, Catroppo JF, Rizvi AA, Powell CK, Cook T, Brown E, Navarro FA, Thornhill J, Burgis J, Jennings WR, McCallum JB, Nottingham JM, Kreiner J, Haddad R, Augustine JR, Pedigo NW, Catalana PV. The evolution of an integrated ultrasound curriculum (iUSC) for medical students: 9-year experience. Crit Ultrasound J 2015;7(1):18. doi: 10.1186/s13089-015-0035-3 26589313 PMC4654731

[18] Rao S, van Holsbeeck L, Musial JL, Parker A, Bouffard JA, Bridge P, Jackson M, Dulchavsky SA. A pilot study of comprehensive ultrasound education at the Wayne State University School of Medicine: a pioneer year review. J Ultrasound Med 2008;27(5):745–9. doi: 10.7863/jum.2008.27.5.745 18424650

[19] Siegel-Richman Y, Kendall J. Establishing an Ultrasound Curriculum in Undergraduate Medical Education: How Much Time Does It Take? J Ultrasound Med. 2018;37(3):569–576. doi: 10.1002/jum.1437 28877363

[20] Russell FM, Zakeri B, Herbert A, Ferre RM, Leiser A, Wallach PM. The State of Point-of-Care Ultrasound Training in Undergraduate Medical Education: Findings From a National Survey. Acad Med 2022;1;97(5):723–727. doi: 10.1097/ACM.0000000000004512 34789665

