# Supplementary material for: SpacED POCUS: A Randomized Controlled Trial of an Adaptive Spaced Education POCUS Curriculum for Medical Students
Source: POCUS J. 2025 Nov 17;10(2):13–9. doi: 10.24908/pocusj.v10i02.19092 (PMC12658577; doi:10.24908/pocusj.v10i02.19092)
Supplement: Supplementary file 1 [file pocusj-10-02-19092-s001.pdf]

Appendix A.

List of pathologies taught and tested

FAST

Presence or absence of free fluid in the right upper quadrant – liver tip, hepatorenal space

Presence or absence of free fluid in the left upper quadrant – spleen tip, spleno-phrenic angle

Presence or absence of free fluid in the pelvis

Presence or absence of pericardial effusion – including trace effusions

For every case, were shown all 5 clips (right upper quadrant, left upper quadrant, subxiphoid OR parasternal long cardiac, transverse bladder/pelvis, longitudinal bladder pelvis) for a FAST exam, to call the exam negative students had to ensure that all 5 clips were indeed negative

False positives: hypoechoic myocardium (false positive for effusion), stomach contents in left upper quadrant, bladder vessels posterior to the bladder, prostate, ovaries, hypoechoic rectal or intestinal intraluminal contents, partially visualized IVC, partially visualized gallbladder, insufficient views of the spleno-phrenic space in the left upper quadrant

Questions were multiple answer/select all that apply. If a student identified free fluid in one quadrant but missed it in another, they got no “partial credit”

Lung

Loss of lung sliding

B lines

Barcode vs seashore sign in M-mode

Spine sign

Lung point

Pneumothorax

Interstitial process

Sub-centimeter consolidation

>1cm consolidation

Pleural effusion

Normal anatomy/artifacts: A lines, Z lines (“comet tails”), rib, normal pleura, normal curtaining/loss of visualization of spine above diaphragm, subcutaneous tissue, diaphragm

Description of findings (e.g. hepatization, airbronchogram, effusion) was separated from interpretation (e.g. pneumothorax, consolidation). Questions emphasized that aside from pneumothorax in a trauma setting, diagnosis was not equivalent to interpretation (e.g. interstitial process does not equal pulmonary edema or heart failure; consolidation could be due to asthma or alveolar hemorrhage or malignancy etc. and not necessarily pneumonia).

Cardiac

Pathologies and normal views were shown in the following views: Parasternal long axis, parasternal short axis, apical four chamber, subxiphoid, IVC

Normal left ventricular function

Left ventricular dysfunction

Right ventricular strain

Cardiac standstill

Pericardial effusion

Normal IVC

Plethoric IVC

Collapsible IVC

Questions were multiple answer/select all that apply. If a student selected a pericardial effusion but did not select LV function/dysfunction, they got no "partial credit."

Vascular

Abdominal aorta, aneurysmal (>3cm diameter)

Abdominal aorta, non-aneurysmal

Abdominal aorta, indeterminate

Common femoral vein, Noncompressible

Saphenofemoral junction, Noncompressible

Popliteal vein, Noncompressible

Superficial peripheral vein, Noncompressible

Indeterminate

False positives: lymph node, bakers cyst, failure to compress vein completely
